# Resveratrol therapeutics combines both antimicrobial and immunomodulatory properties against respiratory infection by nontypeable *Haemophilus influenzae*

**DOI:** 10.1038/s41598-017-13034-7

**Published:** 2017-10-16

**Authors:** Begoña Euba, Nahikari López-López, Irene Rodríguez-Arce, Ariadna Fernández-Calvet, Montserrat Barberán, Nuria Caturla, Sara Martí, Roberto Díez-Martínez, Junkal Garmendia

**Affiliations:** 1 Centro de Investigación Biomédica en Red de Enfermedades Respiratorias (CIBERES), Madrid, Spain; 2 Instituto de Agrobiotecnología, CSIC-Universidad Pública Navarra-Gobierno Navarra, Mutilva, Spain; 3 Facultad de Veterinaria, Universidad de Zaragoza, Zaragoza, Spain; 4 Monteloeder, Elche Parque Empresarial, Elche, Alicante Spain; 5 Departamento Microbiología, Hospital Universitari Bellvitge, University of Barcelona, IDIBELL, Barcelona, Spain; 6 Ikan Biotech SL, The Zebrafish Lab, Centro Europeo de Empresas e Innovación de Navarra (CEIN), Noáin, Spain

## Abstract

The respiratory pathogen nontypeable *Haemophilus influenzae* (NTHi) is an important cause of acute exacerbation of chronic obstructive pulmonary disease (AECOPD) that requires efficient treatments. A previous screening for host genes differentially expressed upon NTHi infection identified sirtuin-1, which encodes a NAD-dependent deacetylase protective against emphysema and is activated by resveratrol. This polyphenol concomitantly reduces NTHi viability, therefore highlighting its therapeutic potential against NTHi infection at the COPD airway. In this study, resveratrol antimicrobial effect on NTHi was shown to be bacteriostatic and did not induce resistance development *in vitro*. Analysis of modulatory properties on the NTHi-host airway epithelial interplay showed that resveratrol modulates bacterial invasion but not subcellular location, reduces inflammation without targeting phosphodiesterase 4B gene expression, and dampens β defensin-2 gene expression in infected cells. Moreover, resveratrol therapeutics against NTHi was evaluated *in vivo* on mouse respiratory and zebrafish septicemia infection model systems, showing to decrease NTHi viability in a dose-dependent manner and reduce airway inflammation upon infection, and to have a significant bacterial clearing effect without signs of host toxicity, respectively. This study presents resveratrol as a therapeutic of particular translational significance due to the attractiveness of targeting both infection and overactive inflammation at the COPD airway.

## Introduction

Though typically a commensal of the nasopharynx, the Gram negative bacterium *H. influenzae*, especially in its noncapsulated or nontypeable form (NTHi), is also an opportunistic pathogen causing middle ear infections (otitis media), conjunctivitis, community-acquired pneumonia, exacerbations of chronic obstructive pulmonary disease (COPD) and, occasionally, invasive disease^1,2^.

COPD is an airway inflammatory disease characterized by a progressive and not fully reversible airflow limitation accompanied by emphysema, fibrosis, chronic overactive inflammation and mucus hypersecretion^3^. COPD is a main leading cause of death, is predicted by the World Health Organization to become the fifth most significant contributor to the worldwide burden of disease by 2020, and it greatly contributes to the economic burden of healthcare costs^4^. Although cigarette smoking is the most common instigating factor in the development of COPD, bacterial colonization of these damaged airways may contribute to disease progression, being NTHi the most common colonizing bacterium in COPD^5^. Moreover, the chronic course and evolution of COPD is often characterized by periods of symptom exacerbation caused by respiratory pathogens such as NTHi (acute exacerbation of COPD, i.e. AECOPD), with a negative impact on the patient’s quality of life and evolution of the disease^6,7^.

Despite the routine therapeutic use of antibiotics, NTHi persists and recurs at the COPD airways, and the numbers of antibiotic-resistant strains increase. NTHi is a facultative intracellular pathogen^8–16^, and subcellular location may allow bacterial cells to temporarily evade the immune system and/or antibiotic-based therapeutic interventions^17,18^. Moreover, given its inflammatory properties, common pharmaceuticals used to manage the symptoms of COPD include β2-agonists, inhaled corticosteroids and phosphodiesterase 4 (PDE4) inhibitors^4,19^. Of note, NTHi infection is also an inflammatory process, and those pharmaceuticals may influence the infectious process^20,21^, as observed for glucocorticoids such as dexamethasone, which attenuate NTHi-triggered inflammation but may also compromise bacterial clearance in mice^22^, or for nonbactericidal PDE4 inhibitors such as roflumilast N-oxide or rolipram, which dampen NTHi-triggered inflammation^23^, dampen NTHi intracellular invasion and enhance mice lung clearance^24^, but may also synergize with NTHi to up-regulate PDE4B2 expression therefore contributing to chemokine induction^25^. Together, success of current therapeutics is limited due to resistance, adverse side effects, tolerance and/or cost^4^. Therefore, novel treatments being safe, effective and not vulnerable to developing resistance are needed to counteract NTHi respiratory infection.

Previous global expression profiling revealed SIRT1, encoding the NAD-dependent deacetylase sirtuin 1, as a host cell gene differentially expressed upon NTHi infection^24^. Emphysema relates to accelerated aging of the lungs, and accelerated aging might be due to defective function of endogenous antiaging molecules such as sirtuins^3^. SIRT1 is a negative regulator of matrix metalloproteinase-9^26^ and protects against emphysema^27^, but its expression is reduced in the peripheral lungs of patients with COPD^26^. Thus, SIRT1 activation may be an attractive therapeutic approach for COPD^28^. Of note, the SIRT1 activator resveratrol (3,5,4´-trihydroxystilbene), a naturally occuring polyphenolic phytoalexin produced by a variety of flowering plants in response to unfavourable environmental conditions and found in dietary products including peanuts, grapes and red wine, reduces NTHi viability^24,29^. Besides NTHi, resveratrol antimicrobial effect has been shown for several Gram positive and negative bacteria^30–33^. Next to its proposed geroprotector role^3,28^, pathway candidates affected by resveratrol exposure in humans are anti-inflammatory mechanisms, cell cycle and programmed cell death pathways, calorie restriction mimetic via energy-sensing metabolic regulators, and anti-oxidant properties^34^. In fact, evidence supports a protective role of resveratrol in respiratory disease as an anti-inflammatory and antioxidant agent^35^. Mechanistically, resveratrol targets are molecules that bind directly with the polyphenol and whose activity, structure, and/or stability is altered as a consequence, or molecules whose expression or activity is altered via an indirect mechanism^34^.

Besides its antimicrobial effect, resveratrol anti-inflammatory action is known to occur during NTHi infection by suppression of extracellular signal-regulated kinases (ERK)-mediated down-regulation of an alternative spliced variant of the adaptor protein myeloid differentiation factor 88 (MyD88) named MyD88 short (MyD88s)^36^. However, both resveratrol antimicrobial and immunomodulatory therapeutic properties have not been jointly considered. The existing body of evidence prompted us to hypothesize that resveratrol effectiveness for the treatment of NTHi respiratory infection may rely on the combined action of its antimicrobial and host cell modulatory effects. Based on this notion, we further analyzed resveratrol antimicrobial properties, its effect on bacterial intracellular location and host gene expression in infected cells, and evaluated resveratrol therapeutic potential *in vivo*. Here we show that resveratrol (i) is bacteriostatic and does not induce resistance development in NTHi, (ii) reduces NTHi airway epithelial invasion but does not alter its subcellular location, (iii) has an anti-inflammatory effect without targeting PDE4B gene expression and dampens human β defensing-2 (hBD2) gene expression in NTHi infected airway epithelial cells, (iv) decreases NTHi viability and reduces airway inflammation in lung infected mice, and (v) has a bacterial clearing effect without signs of host toxicity in a newly established septicemia model system by zebrafish infection with NTHi. Together, this work presents preclinical evidence for resveratrol therapeutic potential against NTHi infection.

## Results

### Resveratrol effect on NTHi is bacteriostatic and does not induce resistance

**Figure 1:**
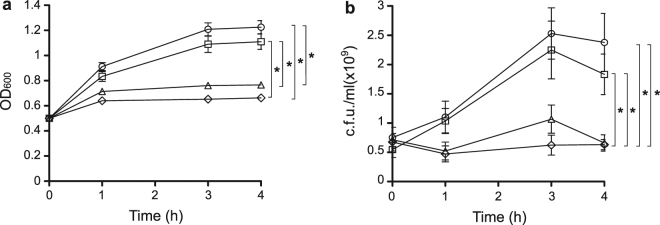
Resveratrol has a bacteriostatic effect on pre-grown NTHi cells. OD_600_-normalized sBHI cultures of NTHi375 were divided into control untreated (circle); resveratrol 112.5 μg/ml (diamond); resveratrol 56.25 μg/ml (triangle); vehicle solution (square), and incubated for 4 h. At the indicated time points, OD_600_ was recorded **(a)**, and samples were serially diluted and plated on sBHI agar for c.f.u./ml determination **(b)**. After 4 h, NTHi375 cells treated with both resveratrol 56.25 and 112.5 μg/ml showed a reduction in OD_600_ and in the number of viable cells compared to control untreated (p < 0.0001) and vehicle solution cultures (p < 0.0001 and p < 0.001, respectively) cultures. Both OD_600_ and number of viable cells were maintained through time in resveratrol treated cultures.

Exposure of NTHi strain 375, hereafter NTHi375, cells in sBHI to increasing concentrations of resveratrol previously revealed bacterial growth inhibition^24^. Resveratrol has been shown to be bacteriostatic on *E. coli* and *Bacillus subtilis* ^31,32^. To evaluate if resveratrol is bactericidal or bacteriostatic on NTHi, we incubated pre-grown NTHi375 cells with resveratrol 112.5 or 56.25 μg/ml (concentrations selected based on previous observations^24^), and monitored the OD_600_ and number of viable cells by serial dilution and plating at the indicated time intervals. After 4 h, resveratrol 112.5 or 56.25 μg/ml-treated cultures showed lower OD_600_ and number of viable cells than the control untreated and vehicle solution ones. Moreover, results of both time and dose dependent assays did not show a reduction in OD_600_ and in the number of viable cells due to resveratrol treatment through time, suggesting it to be bacteriostatic (Fig. 1). We next assessed the ability of NTHi to become resistant to resveratrol through serial passage of strain NTHi375 in sBHI broth containing increasing polyphenol concentrations. A range of resveratrol inhibitory concentrations consisting of 250, 225, 175, 130, 120 and 112.5 μg/ml were employed. After 15 consecutive overnight passages, no resistant bacteria were isolated (Fig. S1). In summary, resveratrol is likely to have a bacteriostatic effect on NTHi and, under the conditions tested, it does not induce resistance on strain NTHi375.

### Resveratrol has an antimicrobial effect on a panel of NTHi COPD clinical isolates

Genomic heterogeneity is a known feature for NTHi^37^, which may lead to variable resveratrol susceptibility among strains, as shown for other antimicrobials^38^. To further expand our observations on NTHi375, we evaluated the effect of resveratrol on NTHi viability by using a panel of 14 clinical strains isolated from COPD sputum samples rendering different typing profiles by PFGE (data not shown). We found that viability decreased for all strains tested in a resveratrol dose-dependent manner (Table 1). As observed for NTHi375, bacterial survival was less than 50% at a resveratrol concentration of 175 μg/ml; for 5 of the isolates analyzed, bacterial survival was less or equal to 10% when incubated with resveratrol 175 μg/ml (Table 1). These results further support resveratrol antimicrobial effect on NTHi. Given that NTHi375 has been previously used in host-pathogen interaction studies^13–15,24,38^, it was next used to assess resveratrol modulatory effect on the NTHi-host airway interplay.

**Table 1:** Percentage of survival by a panel of 14 NTHi COPD clinical isolates, when incubated with increasing concentrations of resveratrol. Values denoted are mean ± SD.

**NTHi strain**	**Resveratrol (μg/ml)**			
	**175**	**75**	**25**	**8.33**
P617–9224	35 ± 11	62.8 ± 14.6	87.9 ± 16.9	98.9 ± 2
P621–7028	24.6 ± 15.2	56 ± 8.3	72.6 ± 19.1	80.8 ± 19.4
P636–8296	23.4 ± 6.9	47.5 ± 22.9	79.7 ± 21	95.6 ± 5.5
P642–4396	22.4 ± 4.5	46.2 ± 6.4	56.4 ± 11.5	73 ± 16.5
P595–8370	31.5 ± 15.5	65.8 ± 9.8	86.2 ± 14.8	90 ± 11.5
P615–8618	11 ± 8	40.3 ± 11.7	52.2 ± 16.5	82 ± 18.5
P650–8603	10.8 ± 4.3	50.8 ± 15.4	81.7 ± 18.3	95.5 ± 7.2
P652–8881	4.8 ± 9.5	42.5 ± 18.4	73.7 ± 19.5	88.3 ± 16.4
P662–7189	15 ± 8.4	40 ± 5.9	61.1 ± 11.7	79.2 ± 12.1
P657–8759	23.6 ± 12.3	60 ± 18.9	83.1 ± 16.3	86 ± 14.1
P665–7858	1.1 ± 2.7	70.7 ± 25	90.1 ± 17	88.5 ± 17.5
P669–6977	24 ± 8.9	57.8 ± 9.6	76.7 ± 10.7	89.8 ± 10.4
P672–7661	2.6 ± 3.2	34.3 ± 10.2	87.4 ± 14.5	96.6 ± 6.8
P679–2791	38.4 ± 10.6	62.4 ± 20.3	74.9 ± 17.8	87 ± 13.2

### Resveratrol efficacy against NTHi infection of cultured airway epithelial cells

In agreement with our previous host expression profiling^24^, NTHi infection increased SIRT1 protein levels in A549 cells. As expected, resveratrol addition also increased SIRT1 protein levels, as already seen in uninfected cells; a synergistic effect between NTHi infection and resveratrol addition on SIRT1 protein levels was not observed (Fig. S2). Conversely, sirtuin-1 siRNA did not modify NTHi epithelial invasion^24^, suggesting that SIRT1 expression/activity and NTHi cell entry may be uncoupled events.

Resveratrol modulates other molecules within the host cell besides SIRT1^39^. Intracellular cAMP increase has been reported to reduce NTHi cell invasion^13,24^. Given that resveratrol inhibits cAMP-degrading PDEs leading to elevated cAMP levels^40^, host cell treatment with this polyphenol should be likely to reduce bacterial infection. However, previous evaluation of resveratrol effect on NTHi airway epithelial infection, performed by A549 cell pre-treatment with resveratrol 20 μM and drug removal before infection, rendered comparable NTHi375 infection levels for both control and resveratrol pre-treated cells^24^. Such assays were performed with resveratrol concentrations reducing bacterial viability, and drug removal prior to infection was necessary to allow quantifying infection rates, which could limit observing a potential resveratrol effect on bacterial location upon infection.

**Figure 2:**
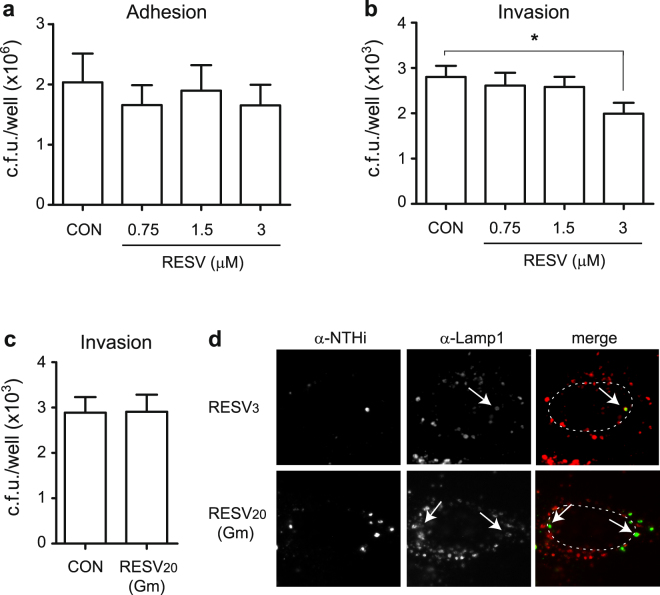
A resveratrol sub-inhibitory concentration lowers NTHi375 invasion of A549 human respiratory epithelial cells. Controls (CON): in adhesion and invasion assays (**a** and **b**, respectively), cells did not receive resveratrol (RESV), but did receive vehicle solution (DMSO) prior and during bacterial contact; in invasion assay (**c**), cells received vehicle solution during gentamicin treatment. Effect of resveratrol sub-inhibitory concentrations on NTHi375 adhesion to- **(a)** and invasion of **(b)** A549 cells. Cells were pre-treated with resveratrol 0.75, 1.5, or 3 μM for 4 h, and the polyphenol was maintained during infection. Bacterial adhesion was comparable for resveratrol-treated and control (CON) cells. Mean numbers of NTHi375 entry into cells treated with resveratrol 3 μM were significantly lower than those obtained for control (CON) cells (p < 0.05). **(c)** Resveratrol effect on intracellular NTHi375. A549 cells were infected and resveratrol 20 μM was added during cell incubation with gentamicin (Gm). Resveratrol did not reduce the number of intracellular bacteria. **(d)** Subcellular location of NTHi375 inside A549 cells shows co-localization with Lamp-1 endosomal marker. NTHi was stained with rabbit α-NTHi and Alexa 488-conjugated donkey α-rabbit (green) antibodies. Lamp-1 was stained with mouse α-Lamp-1 and donkey α-mouse conjugated to RRX (red) antibodies. A549 cells were pre-treated with resveratrol 3 μM for 4 h, which was maintained during infection (upper panels); alternatively, A549 cells were infected for 2 h and resveratrol 20 μM was added during cell incubation with Gm (lower panels). Representative images are shown, taken at 1 h post-Gm. Host cell nuclei location is indicated with dashed lines. Bacteria:Lamp-1 co-localization is indicated with arrows.

Following this notion, we assessed NTHi adhesion to- and invasion of A549 cells pre-treated with resveratrol sub-inhibitory concentrations maintained during bacterial-cell contact. Bacterial adhesion to A549 cells treated with increasing resveratrol concentrations (0.75; 1.5; 3 μM) was comparable to that observed for control untreated cells (Fig. 2a). Differently, a significant decrease on NTHi375 invasion was observed upon cell treatment with resveratrol 3 μM (0.68 μg/ml) (p < 0.05) (Fig. 2b); this resveratrol concentration did not affect NTHi375 bacterial or A549 cell viability (data not shown).

Resveratrol penetration into host cells may occur by a passive diffusion mechanism^41^, and antimicrobial molecules likely to penetrate epithelial cells can reduce intracellular NTHi viability^38^. We next asked if resveratrol could reduce the viability of intracellular bacteria. To do so, A549 cell infection with NTHi375 was performed in culture medium without drug supplementation, followed by subsequent incubation in fresh medium supplemented with gentamicin to kill extracellular bacteria, and without (CON) or with resveratrol 20 μM. Under these conditions, resveratrol did not reduce the number of intracellular bacteria (Fig. 2c). We and others have previously described that NTHi invades epithelial cells and locates inside a non-proliferative compartment with late endosome features^10,13–15^. In agreement, resveratrol pre-treatment or addition during the gentamicin incubation period did not alter such subcellular location (Fig. 2d).

Together, these results show that resveratrol sub-inhibitory concentrations reduce NTHi375 airway epithelial cell invasion and, under the conditions tested, this polyphenol does not modify intracellular bacterial numbers and/or subcellular location.

### Resveratrol modulates NTHi-induced IL-8 and hBD2 expression on A549 airway epithelial cells

**Figure 3:**
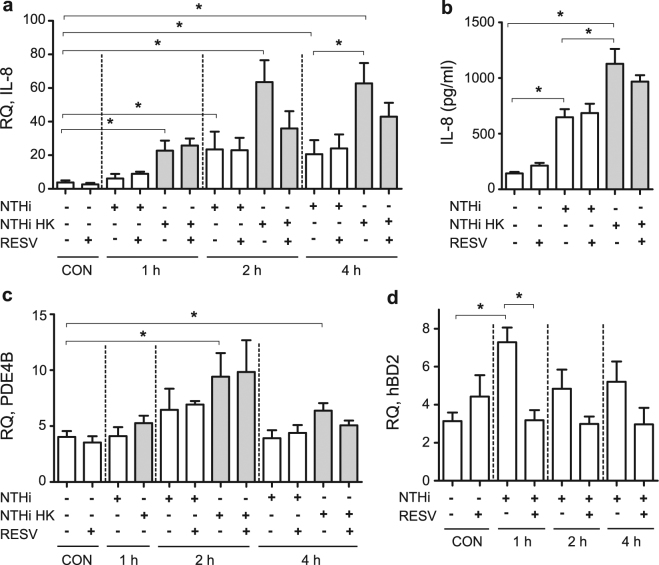
Resveratrol modulates NTHi-induced A549 cell gene expression. Controls (CON): non-infected cells. A549 cells were treated with resveratrol (RESV) 20 μM or DMSO for 4 h, removed prior to bacterial addition (NTHi), or maintained when HK bacteria were used as an inflammatory stimulus (NTHi HK). To monitor gene expression, bacteria were added for 1, 2 or 4 h. Relative quantity (RQ) of human IL-8 (2^−ΔCt^ × 100) **(a)**, PDE4B (2^−ΔCt^ × 100) **(c)**, and hBD2 (2^−ΔCt^ × 1000000) **(d)** mRNA were measured by qRT-PCR. **(b)** Quantification of IL-8 protein secretion by A549 cells, measured by ELISA at 8 h post-infection (hpi). NTHi375 induced IL-8 expression in A549 cells, being significant at 1 (for HK stimulated cells, p < 0.0005), 2 (for NTHi and HK stimulated cells, p < 0.01 and p < 0.0001, respectively) and 4 hpi (for NTHi and HK stimulated cells, p < 0.005 and p < 0.0001, respectively). IL-8 expression was higher in A549 cells incubated with HK bacteria than in NTHi infected cells (for 4 hpi, p < 0.05). NTHi375 induced PDE4B expression in A549 cells, being significant at 2 and 4 hpi (for HK incubated cells, p < 0.005 and p < 0.05, respectively). NTHi375 induced hBD2 expression at 1 hpi (p = 0.001). NTHi triggered IL-8 secretion by A549 cells at 8 hpi (for NTHi and HK stimulated cells, p < 0.0005). IL-8 secretion was higher in cells incubated with HK bacteria than in NTHi infected cells (p < 0.05). Under these conditions, resveratrol shows a trend to reduce IL-8 gene expression and protein secretion, and lowers hBD2 gene expression (p < 0.01). White bars correspond to non-infected (CON) or to NTHi infected cells (NTHi); gray bars correspond to cells stimulated with HK bacteria (NTHi HK).

Despite previous reports on resveratrol antimicrobial and immunomodulatory properties upon NTHi infection^24,36^, the therapeutic potential of this dual effect has not been jointly considered. Thus, resveratrol has been shown to reduce NTHi-induced expression of IL-1β, IL-6, CCL-2 and GM-CSF proinflammatory cytokines in BEAS-2B bronchial epithelial cells^36^. Separately, IL-8 enhanced expression has been observed in NTHi infected A549 cells^24^. Resveratrol effect on NTHi-induced expression of IL-8 in A549 cells was next assessed by cell pre-treatment with resveratrol 20 μM and drug removal before infection, which did not render an anti-inflammatory effect at the gene expression or protein secretion levels (Fig. 3b). This lack of effect could be related to resveratrol removal prior to infection. To maintain resveratrol at the onset of the infectious inflammatory stimulus without jeopardizing bacterial viability, we assessed IL-8 expression and secretion by A549 cells incubated with heat killed (HK) bacteria. Under these conditions, IL-8 expression and secretion were stimulated, compared to control non-infected cells. Indeed, IL-8 expression/secretion levels were higher in cells incubated with HK bacteria than in NTHi infected cells. Moreover, IL-8 gene expression showed a non statistically significant but reproducible trend to be lower in resveratrol-treated cells at 2 and 4 h post-incubation with HK bacteria (Fig. 3a); likewise, IL-8 protein secretion showed the same decreasing trend in resveratrol-treated cells at 8 h post-incubation with HK bacteria (Fig. 3b), compared to control cells receiving vehicle solution.

Resveratrol is known to directly inhibit PDE1, 3 and 4 by competitive inhibition with cAMP in their binding sites^40^. Resveratrol anti-inflammatory effect on NTHi-infected cells may be mediated by MKP-1 phosphatase dependent up-regulation of MyD88s expression via a cAMP-PKA-dependent mechanism^36^. Given that NTHi airway epithelial infection stimulates differential expression for several PDEs including PDE4B (the major PDE isoform expressed in lung)^24^, we asked if resveratrol anti-inflammatory effect on NTHi-infected cells could be also mediated by modulation of NTHi-induced PDE4B gene expression. PDE4B gene expression was assessed in A549 infected cells, in the absence or presence of resveratrol 20 μM. As previously stated, resveratrol removal prior- or maintenance during infection was dependent on the use of intact or HK bacteria, respectively. As shown in Fig. 3c, PDE4B gene expression was increased in NTHi infected- when compared to control uninfected cells (in cells incubated with HK bacteria for 2 and 4 h, p < 0.005 and p < 0.05, respectively). Under these conditions, resveratrol treatment did not modify PDE4B gene expression.

Bacteria-induced human β-defensin-2 (hBD2) antimicrobial peptide expression in lung epithelial cells has also been shown to be differently modulated by resveratrol^42,43^. Based on this notion, we investigated whether NTHi could induce the expression of hBD2 in A549 cells. hBD2 gene expression was higher in NTHi infected than in control uninfected cells (for 1 h infected cells, p = 0.001). Such hBD2 gene expression was lowered to control levels upon resveratrol cell pre-treatment (for 1 h infected cells, p < 0.01) (Fig. 3d). HK bacteria did not modify hBD2 expression on A549 cells, therefore excluding evaluation of a resveratrol-mediated effect under these conditions (data not shown).

These results show that, under the conditions tested, resveratrol anti-inflammatory effect is observed when maintained at the onset of the infectious inflammatory stimulus. Resveratrol treatment of NTHi-infected lung epithelial cells lowers IL-8 gene expression and protein secretion, hBD2 gene expression, and does not interfere PDE4B gene expression.

### Antimicrobial and anti-inflammatory effects of resveratrol administration on mouse pulmonary infection with NTHi

**Figure 4:**
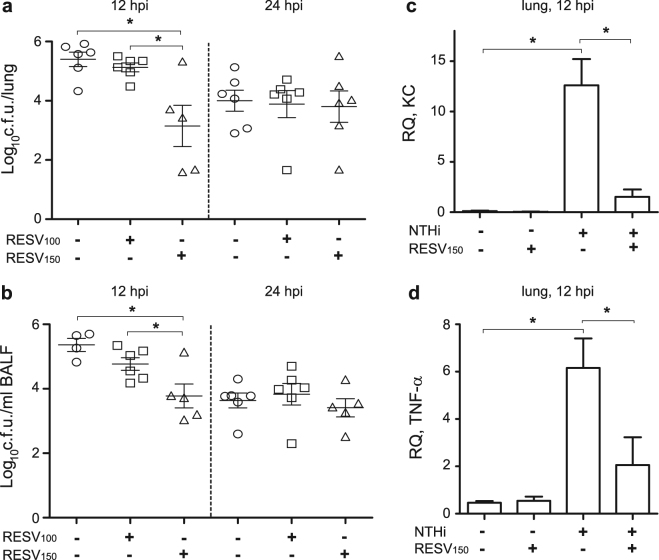
Effect of resveratrol administration on bacterial loads and pro-inflammatory markers in CD1 mice infected by NTHi. Mice were infected intranasally with ∼10^8^ bacteria/mouse. Resveratrol (100 or 150 mg/kg/dose) was administered orally (RESV_100_ or RESV_150_). Controls: animals were administered vehicle solution but did not receive resveratrol. Bacterial counts were determined at 12 and 24 hpi for lungs (log_10_ c.f.u./lung) **(a)** and BALF (log_10_ c.f.u./ml BALF) samples (**b**). At 12 hpi, NTHi375 counts were significantly lower in mice treated with resveratrol 150 mg/kg (triangle) than in control mice treated with vehicle solution (circle) (for lung, p < 0.01; for BALF, p = 0.01), and than in mice treated with resveratrol 100 mg/kg (square) (for lung, p = 0.01; for BALF, p < 0.05). Relative quantities of mouse KC (2^−ΔCt^ × 100) **(c)** and TNF-α (2^−ΔCt^ × 100) **(d)** mRNA were measured by RT-qPCR analysis on lung samples corresponding to non-infected untreated, non-infected resveratrol 150 mg/kg treated, NTHi375 infected untreated, and NTHi375 infected resveratrol 150 mg/kg treated groups. At 12 hpi, KC and TNF-α gene expression was increased in infected compared to non-infected mice (for KC and TNF-α, p = 0.01). Both KC and TNF-α gene expression was lower in NTHi infected resveratrol treated- than in untreated mice (for KC, p < 0.01, for TNF-α, p < 0.05).

Next, we sought to determine the impact of resveratrol oral administration *in vivo* by using a mouse model system of NTHi respiratory infection. We used two regimens of oral resveratrol (100 or 150 mg/kg) consisting of three administrations prior to infection (48, 24, 1 h before infection) and three administrations at 6, 12 and 18 h post-infection (hpi). Fewer NTHi375 bacteria were recovered at 12 hpi from lung and BALF samples of mice treated with resveratrol 150 mg/kg than from mice receiving vehicle solution (for lung, p < 0.01; for BALF, p = 0.01), or receiving resveratrol 100 mg/kg (for lung, p = 0.01; for BALF, p < 0.05) (Fig. 4a,b). Bacterial loads on lung and BALF samples were comparable for resveratrol 100 mg/kg-treated and untreated infected mice. No differences in terms of bacterial counts were observed at 24 hpi among the three conditions tested. Treatment with resveratrol has been shown before to decrease NTHi-induced expression of IL-1β and IL-6 in mice lung tissue^36^. In this study, analysis of NTHi-induced expression of KC and TNF-α pro-inflammatory mediators in mice lung tissue euthanized at 12 hpi rendered higher numbers than those obtained for control non-infected animals (for KC and TNF-α, p = 0.01); moreover, treatment with resveratrol 150 mg/kg decreased NTHi-induced expression of KC and TNF-α in infected mice lung tissue at 12 hpi (for KC, p < 0.01; for TNF-α, p < 0.05) (Fig. 4c,d). No differences in terms of KC and TNF-α expression were observed in resveratrol treated and control untreated non-infected animals.

Microscopy score of average histopathological lesions in samples from mice infected with NTHi375 was also determined along the respiratory tract and compared for untreated and resveratrol 150 mg/kg-treated mice, euthanized at 12 hpi. Overall comparison of scored lesions in the lower airways of NTHi infected mice showed lower inflammatory reaction scores for resveratrol-treated mice. This reduction was significant for PMNs at the bronchi and alveoli in infected mice treated with resveratrol, compared to control untreated mice (p = 0.01) (Table 2). As an exception, the upper airways of resveratrol-treated infected mice showed a significant increase for PMNs at the nasal cavity compared to control untreated animals (p < 0.05) (Table 2).

**Table 2:** Score of histopathological lesions found in the airways of untreated or resveratrol treated mice, intranasally infected with NTHi375.

		^**b**^ **Score (mean** ± **SD)**							
		**Upper airways**				**Lung**			
**Infecting NTHi strain**	^**a**^ **Treatment**	**Secretion and erythrocytes in lumen**	**PMNs lumen**	**Hyperemia**	**PMNs lamina propria**	**Haemorrhage**	**Alveolar macrophages**	**Hyperemia**	**Bronchial-alveolar PMNs**
NTHi375	Control untreated	1.7 ± 0.4	1.6 ± 0.2	1 ± 0.2	1.4 ± 0.2	0.2 ± 0.2	0.5 ± 0	0.7 ± 0.1	0.9 ± 0.1
NTHi375	RESV_150_	1 ± 0.3	^c^2.5 ± 0.2	0.8 ± 0.1	1 ± 0.2	0.1 ± 0.1	0.5 ± 0	0.8 ± 0.1	^d^0.3 ± 0.1

^a^Control untreated animals were administered vehicle solution (PBS:DMSO, 1:1). RESV treated animals were administered three RESV doses (150 mg/kg) at 48, 24, and 1 h before infection and one RESV (150 mg/kg) dose at 6 hpi. Mice were euthanized at 12 hpi. ^b^Statistical comparisons of mean values were performed using the two-tail *t* test. ^c^Larger numbers of PMNs in the upper airways lumen of RESV treated than control untreated animals (p < 0.05) infected by NTHi375. ^d^Lower PMNs numbers at the bronchi and alveoli of mice treated with RESV, compared to controls (p =0.01) infected by NTHi375.

In sum, these results show that resveratrol 150 mg/kg reduces bacterial load and whole-lung inflammatory markers such as KC and TNF-α. This is in turn reflected by lower PMN in the broncho-alveolar space.

### Resveratrol has an antimicrobial protective effect on zebrafish infection with NTHi

Zebrafish has been used for over a decade to study the mechanisms of a variety of inflammatory disorders and infectious diseases. Zebrafish presents adaptive immunity 4–6 weeks after birth, when it becomes a suitable model to analyze novel antimicrobial agents^44^. Thus, aiming to validate the *in vivo* data shown above in an alternative animal model of infection, we tested *H. influenzae* infection on adult zebrafish by bacterial intraperitoneal injection^45,46^. The infection dose was first optimized by injection of 6 animal groups (n = 10) with 10 μl of a NTHi375 suspension containing 5 × 10^5^, 5 × 10^6^, 5 × 10^7^, 5 × 10^8^ or 5 × 10^9^ c.f.u./ml, or saline solution 0.9% as a control. NTHi375 severely reduced zebrafish survival. An infection dose consisting of ∼5 × 10^7^ c.f.u./zebrafish caused progressive death of the animals after injection (data not shown), therefore presenting a novel preclinical sepsis model system suitable for further use.

**Figure 5:**
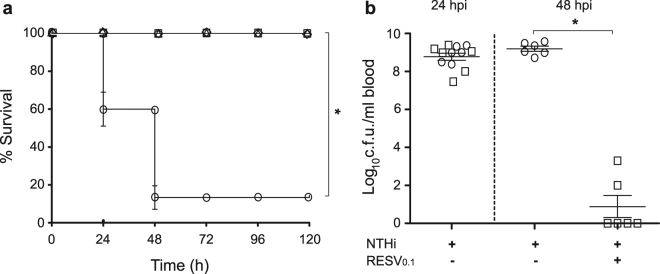
Effect of resveratrol administration on zebrafish infected by NTHi. Zebrafish were infected intraperitoneally with ∼5﻿×﻿10^7^ bacteria/individual. When necessary, resveratrol 0.1 mg/g/dose (RESV_0.1_) was administered intraperitoneally at 29 and 53 hpi. **(a)** Effect of resveratrol administration on adult zebrafish survival upon NTHi375 infection. Non-infected groups were administered perfusion solution-DMSO (1:1) (triangle) or saline solution 0.9% (diamond); infected groups were administered perfusion solution-DMSO (1:1) (circle) or resveratrol (square). Survival rate is reported as percentage (mean ± SD) of adult individuals survival at 120 hpi. Survival of NTHi infected zebrafish was significantly higher in resveratrol treated- and in untreated animals (p < 0.0001). **(b)** Bacterial counts on zebrafish blood samples from the caudal fin were determined at 24 and 48 hpi (log_10_ c.f.u./ml blood), corresponding to 5 h before- and 19 h post-resveratrol treatment, respectively. At 48 hpi, NTHi375 counts were lower in resveratrol treated (square) than in control vehicle administered (circle) zebrafish (p < 0.0001). Perfusion solution-DMSO (1:1) was used as vehicle solution.

Before performing a resveratrol protective assay in adult zebrafish, a resveratrol acute toxicity assay was carried out in zebrafish embryos following the OECD TG 236 “Fish embryo acute toxicity (FET) test”. Results showed that resveratrol was not toxic, being 1 mg/ml the highest dose tested (data not shown). Next, we assessed resveratrol antimicrobial effect on NTHi375 infected zebrafish by using a therapeutic regimen of intraperitoneal resveratrol (0.1 mg/g) consisting of two administrations at 29 and 53 hpi. Survival rate for resveratrol-treated and control untreated groups was monitored up to 5 days post-infection. Mortality rate in resveratrol-treated infected zebrafish was significantly lower than in control animals receiving vehicle solution (p < 0.0001); indeed, survival was similar for infected zebrafish receiving resveratrol than for control non-infected animals (Fig. 5a). To quantify resveratrol protective effect, we determined the number of bacterial c.f.u. by collecting blood from the caudal fin of NTHi infected zebrafish at 1 and 2 days post-challenge, corresponding to 5 h before- and 19 h post-resveratrol treatment. Significantly fewer NTHi375 bacteria were recovered at 19 h post-treatment from zebrafish treated with resveratrol than from those receiving perfusion solution-DMSO (1:1) (p < 0.0001) (Fig. 5b).

Altogether, these results present zebrafish as a preclinical sepsis model system useful for *in vivo* therapeutic evaluation against NTHi infection. By using this system, we observed both significant resveratrol-mediated NTHi clearing effect upon zebrafish infection and subsequent increased survival.

## Discussion

This study delineates the effect of resveratrol administration on infection by NTHi. By using conditions mimicking therapeutic resveratrol administration *in vitro*, in cultured respiratory epithelial cells and *in vivo*, we established the impact of this natural polyphenol on a number of properties related to NTHi viability, host-pathogen interplay and progression of infection. Given that NTHi is largely associated with COPD chronic infection and AECOPD frequency^47^, therapeutics targeting both infection and overactive inflammation at the COPD airway are of particular interest. Preventive and therapeutic benefits of this antimicrobial/anti-inflammatory duality have been exploited for the macrolide antibiotic azithromycin. Thus, clinical studies suggest that azithromycin long-term low-dose use to prevent and/or manage chronic inflammatory airway disorders such as AECOPD may be beneficial, but could also increase the rate of macrolide resistance by colonising opportunistic pathogens. In fact, adverse effects of long-term azithromycin use in patients with chronic lung diseases have been suggested^48^, and its use for AECOPD prevention is under debate. Following this notion, this study assessed resveratrol therapeutic potential against NTHi infection, which may also rely on its antimicrobial/anti-inflammatory duality. Previous work has been separately performed on resveratrol antibacterial and anti-inflammatory activities against NTHi infection^24,36^. This is, to our knowledge, the first study jointly considering the therapeutic benefits of both properties. Thus, our use of *in vitro* and *in vivo* model systems has allowed establishing associations between resveratrol effects, doses, bacterial inhibitory concentrations and antimicrobial/host modulatory properties.

Resveratrol has demonstrated antibacterial activity against several Gram-negative and Gram-positive bacterial pathogens^30–33,49,50^. Mechanistically, it is thought to work by inhibiting bacterial ATP synthase^51^, and by site-specific oxidative damage to the bacterial cell, resulting in a bacteriostatic effect^31,32^. Our results show that resveratrol is likely to be bacteriostatic on NTHi, it has a comparable effect on a whole range of genomically unrelated NTHi clinical isolates, and resistance is not induced *in vitro*; as a counterpoint to these likely beneficial therapeutic properties, and different to macrolides^38,52^, resveratrol does not seem to reduce NTHi intracellular numbers despite using working concentrations shown to reduce viability for the infecting strain.

At the cellular level, our results indicate that each experimental setting is crucial to properly address resveratrol effects during infection. Thus, resveratrol concentrations inhibiting NTHi viability or being sub-inhibitory determine drug removal prior to- or drug maintenance during infection, respectively. Of note, although evaluation of resveratrol impact on the NTHi-airway epithelia interface by cell pre-treatment with a resveratrol bactericidal concentration did not modify NTHi invasion rate^24^, a maintained sub-inhibitory dose is able to lower NTHi epithelial invasion. We speculate that resveratrol effects could be transitory and/or reversible, and this aspect should be further elucidated and taken into consideration when designing any administration regimen. Consequently, this notion may also apply when assessing resveratrol immunomodulatory effects on infected cells. Resveratrol has been shown to suppress the inflammatory response of host cells infected by several bacterial pathogens including *Porphyromonas gingivalis* and *Streptococcus pneumoniae* ^43,53^, and also to reduce inflammation on NTHi-infected cells^36^; of note, such observation did not consider resveratrol antimicrobial effect therefore maintaining the polyphenol upon cell infection with NTHi strain 12. In practical terms, the use of HK bacteria is likely to mimic the inflammatory stimulus and allow assessing resveratrol immunomodulatory properties on cells infected by susceptible pathogens without jeopardizing their viability. This approach previously allowed addressing resveratrol-based suppression of inflammatory responses on *P. gingivalis* infected human gingival epithelia^53^, and has been used in the present study, showing a trend to reduce IL-8 expression in NTHi HK infected human airway epithelial cells. Resveratrol also modulates positive or negatively antimicrobial peptide (AMP) expression in cells infected by *S. pneumoniae* or *Pseudomonas aeruginosa*, respectively^42,43^. NTHi has been shown to induce AMP expression in primary bronchial epithelial cells^54^, and we show here that resveratrol reduces hBD2 expression on A549 cells infected by NTHi. In addition, resveratrol has anti-oxidant properties in cultured epithelial cells infected by respiratory pathogens inducing oxidative stress, such as *S. pneumoniae* and *P. aeruginosa* ^42,55^. NTHi has been shown to induce lung oxidative stress mainly in phagocytic cells^56^; however, we could not detect this stress in infected cultured epithelia (data not shown), therefore excluding the possibility of assessing a potential resveratrol antioxidant effect on NTHi infected cells. Given that oxidative stress is intimately associated to COPD progression and exacerbation, therapeutic intervention with antioxidants may have a beneficial outcome in the treatment of COPD^57^.

Future work will tackle alternative host cell infection model systems to monitor resveratrol antioxidant properties upon NTHi infection.

Resveratrol effectiveness has been shown by reducing bacterial loads in lungs and BALF samples during NTHi mouse respiratory infection, with a clearing effect dependent on resveratrol dose. Resveratrol antimicrobial potential has been previously proven in rat and mouse models infected by *Serratia marcescens* or respiratory syncytial virus, respectively^58,59^. In this study, we further support resveratrol antimicrobial effect by presenting a newly established preclinical sepsis model system by NTHi zebrafish infection. Moreover, testing resveratrol toxicity in zebrafish supports previous evidences for its low cytotoxicity in human cells^33^. Besides its antibacterial effect, evidence suggests that resveratrol has anti-inflammatory properties *in vivo* ^59,60^. This is also the case in the lungs of NTHi infected mice^36^. Our results revealed parallel bacterial clearance and reduction of inflammation in resveratrol treated NTHi infected lungs. Of note, the upper airways of resveratrol-treated infected mice showed increased PMN numbers at the nasal cavity compared to untreated animals, which could somehow contribute to reduce bacterial load and inflammation at the lower airway, even though the reasons for such increase are currently unknown. Likewise, we acknowledge that the observed suppression of lung inflammation could be not only due to resveratrol anti-inflammatory properties, but also to its bacterial clearing effect, actively reducing the inflammatory stimulus by lowering bacterial viability. Importantly, this aspect should be taken into consideration when analyzing and concluding on potential immunomodulatory properties by antimicrobial therapeutic agents. The use of anti-inflammatory therapeutics against NTHi respiratory infection has been previously tested *in vivo* for dexamethasone, azithromycin and the non-bactericidal PDE4 inhibitors roflumilast N-oxide and rolipram, highlighting the benefits of PDE4 inhibition^22–24,38^. Resveratrol is known to inhibit PDE4, but our PDE4B expression data did not show a resveratrol inhibitory effect at the gene expression level, leading us to speculate that it may occur by competitive inhibition with cAMP, as previously stated^40^.

Overall, plant polyphenols are important components of human diet, and a number of them are considered to possess chemopreventive and therapeutic properties. Numerous dietary plants contain polyphenols, resveratrol belonging to the non-flavonoid family. Although no studies have specifically examined resveratrol concentrations in relation to respiratory disease risk, a number of epidemiologic studies identified that consumption of various antioxidant-rich foods, many of which known to contain resveratrol, are protective against asthma outcomes^35^. Resveratrol-driven inflammatory reduction in COPD^28,61–63^ and its potential to slow aging-related deterioration of lung function and structure by maintaining alveolar cell integrity^64^, support a resveratrol protective role against chronic airway disease. The present study sheds light on resveratrol as an attractive therapy that timely combines anti-inflammatory and antimicrobial properties, therefore targeting both infection and overactive inflammation at the COPD airway. Future research will consider the efficacy and toxicity of resveratrol when present in the human airways, and explore the therapeutic potential of other plant polyphenols against respiratory infection.

## Methods

### Bacterial strains, media, growth conditions, interfering drugs

NTHi strains were grown at 37 °C, 5% CO_2_ on chocolate agar (Biomérieux) or on brain heart infusion (BHI) agar supplemented with 10 μg/ml hemin and 10 μg/ml nicotinamide adenine dinucleotide (NAD), referred to as sBHI. NTHi liquid cultures were grown in sBHI. When necessary, heat killed (HK) bacteria were used; for this purpose, a bacterial suspension recovered with 1 ml PBS from a freshly grown chocolate agar plate was adjusted to OD_600_ = 1 (∼10^9^ c.f.u./ml) and incubated at 80 °C for 30 min. NTHi375 is a genome sequenced clinical isolate previously used in host-pathogen interplay studies^13,15,65,66^. NTHi clinical strains shown Table 1 were isolated from COPD sputum samples during independent AECOPD episodes at Bellvitge University Hospital, Spain. Strain relatedness was determined by pulse field gel electrophoresis (PFGE)^67^. Resveratrol was purchased from Sigma-Aldrich, and was also kindly supplied by Monteloeder (Elche, Spain). Resveratrol (20 mM, i.e. 4.56 mg/ml) stock solutions were prepared in dimethyl sulfoxide (DMSO) and diluted to the required working concentrations in Earle’s balanced salt solution (EBSS; Gibco), sBHI or PBS, depending on the assay type (see below).

### Resveratrol susceptibility assays

NTHi susceptibility to resveratrol was assessed as described before^24^. To assess if resveratrol effect on NTHi is bactericidal or bacteriostatic, bacteria grown on chocolate agar for 16 h were inoculated (2 to 5 colonies) in 20 ml sBHI and incubated for 11 h under shaking. Cultures were then diluted in 100 ml sBHI to OD_600_ = 0.05, incubated with agitation to OD_600_ = 0.5 and divided into 4 flasks (20 ml/flask) as it follows: (i) control untreated; (ii) resveratrol 112.5 μg/ml; (iii) resveratrol 56.25 μg/ml; (iv) DMSO, by adding a volume identical to that used in (ii). Cultures were incubated with agitation for 4 h. When indicated, OD_600_ was recorded and culture samples were serially diluted and plated on sBHI agar. Data are shown as OD_600_ and c.f.u./ml. Experiments were performed in duplicate at least four times (n > 8).

### NTHi serial passage in the presence of resveratrol

Bacteria grown on chocolate agar for 16 h were inoculated (1 colony) in 3 ml sBHI and incubated for 11 h under shaking; starting inocula were prepared in quadruplicate (cultures 1 to 4, CL1 to CL4). Resveratrol 20 mM stock solution was diluted in sBHI (250; 225; 175; 130; 120; 112.5 μg/ml). One hundred μl of each resveratrol dilution were transferred to individual wells in 96-well microtiter plates; a vehicle solution control consisting of a DMSO volume equivalent to that used for the highest resveratrol concentration tested was performed in parallel. The assay was initiated by inoculating 1 μl of the previously grown bacterial cultures in each well, and further incubation for 24 h at 37 °C shaking. Cultures were then passaged (10 μl in 100 μl fresh sBHI with resveratrol or vehicle solution) every day for 15 days. At various time points throughout the cycling, culture samples were serially diluted and plated on sBHI agar. The assay was performed in duplicate (n > 8).

### Infection of cultured epithelial cells

Carcinomic human alveolar basal epithelial cells (A549, ATTC CCL-185) were maintained, and NTHi infected to quantify bacterial adhesion and invasion, as described previously^15^. When indicated, cells were pre-treated with resveratrol (0.75; 1.5; 3 μM) or vehicle solution (DMSO) for 4 h in 1 ml EBSS, and drug exposure was maintained during bacterium-cell contact. Alternatively, cells were infected, and resveratrol 20 μM or DMSO was added during the gentamicin incubation period. These treatments did not induce A549 cytotoxicity, verified by measuring the release of lactate dehydrogenase and by microscopy (data not shown). Controls (CON) were performed by using a DMSO volume corresponding to that of the highest resveratrol concentration tested in each assay. Results are expressed as c.f.u./well. Experiments were performed in triplicate and at least in three independent occasions (n ≥ 9).

### Immunofluorescence microscopy

A549 cells were seeded on 13 mm circular coverslips in 24-well tissue culture plates and NTHi infected for 2 h as previously described^15^. Infected cells were incubated in RPMI 1640 containing 10% FCS, Hepes 10 mM and gentamicin 200 μg/ml for 1 h. When necessary, cells were pre-treated with resveratrol 3 μM or DMSO for 4 h in 1 ml EBSS, maintained during bacterium-cell contact; alternatively, cells were infected, and resveratrol 20 μM or DMSO was added during the gentamicin incubation period. Cells were washed three times with PBS and fixed with 3.7% paraformaldehyde (PFA) in PBS pH 7.4 for 15 min at room temperature. Bacteria were stained with rabbit anti-NTHi serum^15^ diluted 1:600. Late endosomes were stained with mouse monoclonal anti-human Lamp-1 H4A3 antibody (Developmental Studies Hybridoma Bank) diluted 1:70. Donkey anti-rabbit conjugated to Alexa 488 and donkey anti-mouse conjugated to Rhodamine Red-X (RRX) secondary antibodies (Jackson Immunological) were diluted 1:100. Samples were analyzed with a Carl Zeiss Axioskop 2 plus fluorescence microscope and a Carl Zeiss Axio Cam MRm monochrome camera.

### RNA extraction and real-time quantitative PCR

A549 cells were seeded on 24-well tissue culture plates, and infected for 1, 2 or 4 h in EBSS. When indicated, cells were pre-treated with resveratrol 20 μM or DMSO for 4 h in 1 ml EBSS, and removed prior to bacterial addition. Alternatively, cells were pre-treated with resveratrol 20 μM or DMSO for 4 h in 1 ml EBSS, and a suspension containing HK bacteria (equivalent in numbers to that of the infecting dose) was added for 1, 2 or 4 h, without removal of resveratrol or DMSO. Following treatment and/or NTHi stimulation, total RNA was isolated from A549 cells using a Nucleospin RNAII kit (Macherey-Nagel) as recommended by the manufacturer and including an on column DNase treatment step. When necessary, total RNA was isolated from mouse lungs using TRIzol reagent (Invitrogen). Total RNA quality was evaluated using RNA 6000 Nano LabChips (Agilent 2100 Bioanalyzer, Santa Clara, CA). All samples had intact 18 S and 28 S ribosomal RNA bands with RNA integrity numbers (RIN) between 9.3 to 10. Reverse transcription was performed using 1 μg of RNA by PrimerScript RT Reagent kit (Takara). To amplify human *il-8*, *pde4b*, and mouse *kc*, *tnf-α*, *gapdh* genes, 6 ng of cDNA were used; to amplify human *hbd2*, 50 ng of cDNA were used as template. In all cases, 20 μl reaction mixtures containing 1X SYBR Premix Ex Taq II (Tli RNaseH Plus) (Takara) and the adequate primer mix were used. When necessary, human *gapdh* was amplified using 6 or 50 ng of cDNA as template. Fluorescence data were analyzed with AriaMx Real-Time PCR System (Agilent Technologies). The comparative threshold cycle (Ct) method was used to obtain relative quantities of mRNAs that were normalized using human or mouse *gapdh* as an endogenous control. Intron-spanning primers were designed with Primer-BLAST software (NCBI)^24,68^ (Table S1). All measures were performed in triplicate and at least four times (n > 12).

### Western Blot

A549 cells were seeded before each experiment on 6-well tissue culture plates at 2.8 × 10^6^ cells per well. Cells were pre-treated with resveratrol 20 μM for 4 h in 4 ml EBSS per well, and then infected for 10, 20, 30, 50, 70, 90, 100 or 120 min with NTHi375. Next, wells were washed 3 times with cold PBS, scrapped and lysed with 100 μl of lysis buffer (62.5 mM Tris-HCl pH 6.8, 2% w/v SDS, 10% glycerol, 50 mM DTT, 0.01% w/v bromophenol blue) on ice. Samples were sonicated, boiled at 100 °C for 10 min and cooled on ice before 10% SDS-PAGE and western blotting. SIRT1 was detected with primary rabbit anti-SIRT1 antibody (sc-15404, Santa Cruz Biotechnology) diluted 1:1,000; tubulin, used as a loading control, was detected with primary mouse anti-tubulin antibody (Sigma-Aldrich) diluted 1:3,000. Secondary goat anti-rabbit IgG and anti-mouse IgG (whole molecule, Sigma-Aldrich) antibodies, conjugated to horseradish peroxidase, were diluted 1:1,000. ECL AdvanceTM Western Blotting Detection Kit (GE HealthCare) was used for detection. Western blots were performed at least three times by using independently generated A549 cell extracts. Densitometry analysis on scanned images was performed using ImageJ software (http://rsb.info.nih.gov/ij/download.html). Bands in each lane were analyzed using the label peaks tool, and the mean intensity was recorded. Results are expressed as relative level of protein (mean intensity of protein SIRT1/mean intensity of tubulin).

### Secretion of IL-8

A549 cells were maintained, seeded on 24-well tissue culture plates, and infected for 2 h. When necessary, cells were pre-treated with resveratrol 20 μM or DMSO for 4 h in 1 ml EBSS, and removed prior to bacterial addition. Alternatively, cells were pre-treated with resveratrol 20 μM or DMSO for 4 h in 1 ml EBSS, and a suspension containing HK bacteria was added for 2 h, without removal of resveratrol or DMSO. Cells were washed 3 times with PBS and incubated for 6 h in RPMI 1640 containing 10% FCS, Hepes 10 mM, gentamicin 100 μg/ml, and resveratrol 20 μM or DMSO. Supernatants were collected from the wells, cell debris was removed by centrifugation and samples were frozen at −80 °C until use. IL-8 levels in the supernatants were measured by ELISA (Abnova KA0115) with sensitivity <2 pg/ml. Results are expressed as IL-8 pg/ml. Infections were performed in duplicate and at least twice (n > 4).

### NTHi mouse lung infection

A previously described mouse model of NTHi respiratory infection was used^24,38,67,69,70^ at the Institute of Agrobiotechnology facilities (registration number ES/31–2016–000002-CR-SU-US). Animal handling and procedures were in accordance with the current European (Directive 86/609/EEC) and National (Real Decreto 53/2013) legislations, following the FELASA and ARRIVE guidelines, and with the approval of the Universidad Pública de Navarra (UPNa) Animal Experimentation Committee (Comité de Ética, Experimentación Animal y Bioseguridad) and the local Government authorization. Resveratrol treatment was performed at doses of 100 or 150 mg/kg of body weight in 0.1 ml PBS-DMSO (1:1) and administered by oroesophageal gavage (Popper&Sons Inc.). Administrations were performed at 48, 24, 1 h before infection and at 6, 12 and 18 h post-infection (hpi) (resveratrol administrations at 12 and 18 hpi were performed in mice euthanized at 24 hpi). NTHi375 was used for lung infection, and mice were randomly divided into 6 infected (n = 6) and 2 control non-infected (n = 3) groups: (i) treated with 100 mg/kg resveratrol, euthanized at 12 hpi; (ii) treated with 100 mg/kg resveratrol, euthanized at 24 hpi; (iii) treated with 150 mg/kg resveratrol, euthanized at 12 hpi; (iv) treated with 150 mg/kg resveratrol, euthanized at 24 hpi; (v) treated with vehicle solution (PBS-DMSO, 1:1), euthanized at 12 hpi; (vi) treated with vehicle solution, euthanized at 24 hpi; (vii) control, administered 150 mg/kg resveratrol; (viii) control, administered vehicle solution. For NTHi intranasal infection, 20 μl of a NTHi375 suspension containing ∼5 × 10^9^ c.f.u./ml (1 × 10^8^ c.f.u./mouse) was placed at the entrance of the nostrils until complete inhalation by the mouse, previously anesthetized (ketamine-xylazine, 3:1). At 12- or 24 hpi, mice were euthanized and lungs were aseptically removed. The left lung was individually weighed in sterile bags (Stomacher80, Seward Medical) and homogenized 1:10 (wt/vol) in PBS. Each homogenate was serially 10-fold diluted in PBS and plated in triplicate on sBHI agar to determine the number of viable bacteria. Results are shown as log_10_ c.f.u./lung. The right lung was fixed in 10% neutral buffered formalin. Heads and necks, containing upper airways, larynxes, and tracheas, were fixed in the same buffered formalin for histology. In parallel, BALF samples were obtained by perfusion and collection of 0.7 ml of PBS, with the help of a sterile 20 G (1.1-mm diameter) Vialon intravenous catheter (Becton-Dickinson) inserted into the trachea. Each recovered BALF fraction was serially 10-fold diluted and plated on sBHI agar as described above. Results are shown as log_10_ c.f.u./ml BALF.

Histopathology and lesions score was performed as it follows. Heads and necks were rinsed in running tap water for 1 h, immersed in 5% nitric acid for 24–36 h until complete decalcification, and 7–8 transaxial slices were made every 3–4 mm beginning at the nostrils and finishing in the caudal tracheas. Transaxial slices and lungs were embedded in paraffin, and 4- to 6 μm sections were stained with hematoxylin and eosin (H&E) by standard procedures, and examined by microscopy to determine the presence and extent of inflammatory lesions. Sections were examined blind as sets by a trained veterinary pathologist (Dr. M. Barberán). Parameters characterizing an acute inflammatory reaction in upper airways and lung, including hemorrhages, hyperemia, polymorphonuclear cell infiltrates (PMNs) and alveolar macrophages, were subjectively scored on a scale of 0 to 3 (0: absent, 1: mild, 2: moderate, 3: severe). For tissue control, similar organs obtained from non-infected control and resveratrol treated mice were processed in an identical manner to the infected tissues. Images were observed and digitalized using an Olympus Vanox AHBS3 microscope coupled to an Olympus DP12 digital camera.

### NTHi adult zebrafish infection

Animal experiments conducted at The Zebrafish Lab (http://www.thezebrafishlab.com) animal housing facility were performed according to EU guidelines (http://ec.europa.eu/environment/chemicals/lab_animals/home_en.htm), and the approval of the Universidad de Navarra (UNAV) Ethics Committee for Animal Experimentation (Protocol 03417). Once lack of resveratrol toxicity in zebrafish was confirmed (data not shown), adult zebrafish (n = 10 per group) were randomly divided into 2 infected and 2 non-infected groups. Infected groups were injected with 10 μl of a NTHi375 suspension containing ∼5 × 10^9^ c.f.u./ml, i.e. ∼5 × 10^7^ c.f.u./zebrafish, prepared in perfusion solution (Grifols, Spain). At 29 and 53 hpi, an infected group was intraperitoneally administered resveratrol at a dosis of 0.1 mg/g of body weight in 10 μl of perfusion solution-DMSO (1:1); the other groups were administered perfusion solution-DMSO (1:1) or saline solution 0.9% as a control. Survival rate for each group was monitored three times per day during 5 days after infection. To quantify resveratrol protective effect, the numbers of bacterial c.f.u. in blood samples collected from zebrafish caudal fin were quantified at 24 and 48 hpi, i.e. 5 h before- and 19 h post-treatment. Each recovered blood fraction was serially 10-fold diluted in PBS and spread on BHI agar to determine the number of viable bacteria. Results are shown as log_10_ c.f.u./ml blood.

### Statistical analysis

For bacterial viability, cell infection, gene expression, IL-8 secretion and bacterial loads in lungs and BALF samples, mean±SD were calculated and statistical comparisons of means were performed using the two-tail Student’s *t* test. For zebrafish assays, statistical analysis were performed by using the two-tailed Student’s *t* test (for two groups), analysis of variance (ANOVA) was chosen for multiple comparisons, and the log-rank (Mantel–Cox) and Gehan–Breslow–Wilcoxon tests were used to draw, analyze and compare survival curves. In all cases, a p < 0.05 value was considered statistically significant. Analyses were performed using the Prism software, version 7 for Mac (GraphPad Software) statistical package.

## Electronic supplementary material

Supplementary Information
