# Supplementary material for: Resveratrol therapeutics combines both antimicrobial and immunomodulatory properties against respiratory infection by nontypeable Haemophilus influenzae
Source: Sci Rep. 2017 Oct 16;7:12860. doi: 10.1038/s41598-017-13034-7 (PMC5643544; doi:10.1038/s41598-017-13034-7)
Supplement: Supplementary file 1 — Supplementary Information [file 41598_2017_13034_MOESM1_ESM.pdf]

Supplementary information

Resveratrol therapeutics combines both antimicrobial and immunomodulatory properties against respiratory infection by nontypeable *Haemophilus influenzae*

Begoña Euba[1,2+], Nahikari López-López[2+], Irene Rodríguez-Arce[2], Ariadna Fernández-Calvet[2], Montserrat Barberán[3], Nuria Caturla[4], Sara Martí[1,5], Roberto Díez-Martínez[6], Junkal Garmendia[1,2*]

[1]Centro de Investigación Biomédica en Red de Enfermedades Respiratorias (CIBERES), Madrid, Spain; [2]Instituto de Agrobiotecnología, CSIC-Universidad Pública Navarra-Gobierno Navarra, Mutilva, Spain; [3]Facultad de Veterinaria, Universidad de Zaragoza, Spain; [4]Monteloeder, Elche Parque Empresarial, Elche, Alicante, Spain; [5]Departamento Microbiología, Hospital Universitari Bellvitge, University of Barcelona, IDIBELL, Barcelona, Spain; [6]Ikan Biotech SL, The Zebrafish Lab, Centro Europeo de Empresas e Innovación de Navarra (CEIN), Noáin, Spain

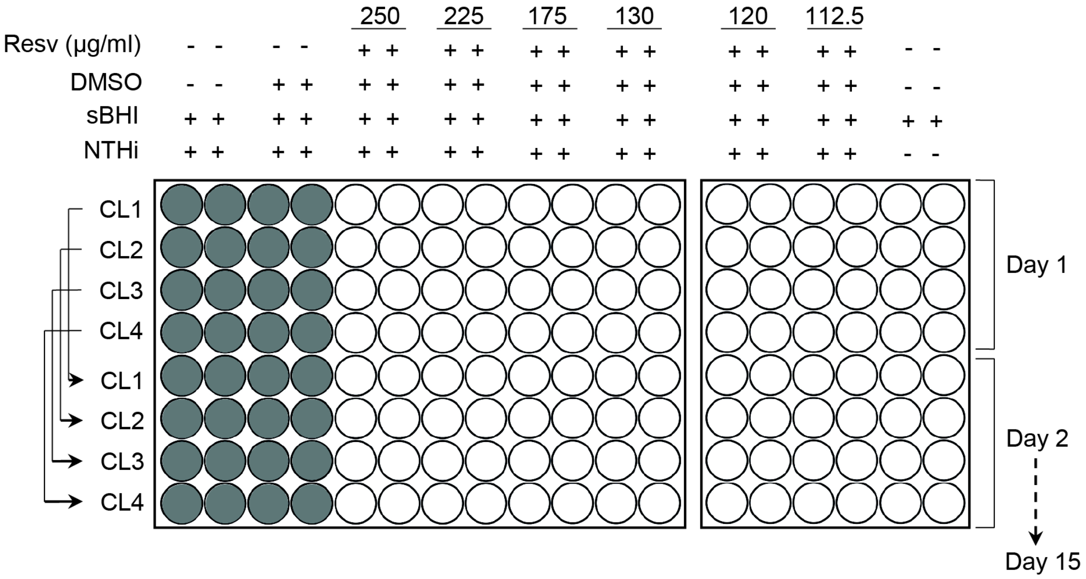

Figure S1. NTHi serial passage in the presence of resveratrol does not induce resistance. Four independently grown NTHi375 cultures (CL), CL1 to CL4, were exposed to a range of resveratrol inhibitory concentrations consisting of 250, 225, 175, 130 and 112.5 µg/ml. sBHI without/with DMSO were used as positive controls for NTHi growth. After 15 serial overnight passages, lack of growth, i.e. no resistant bacteria, was observed under the conditions tested. Lack of growth is represented by white empty wells; turbidity and growth is represented by gray filled wells.

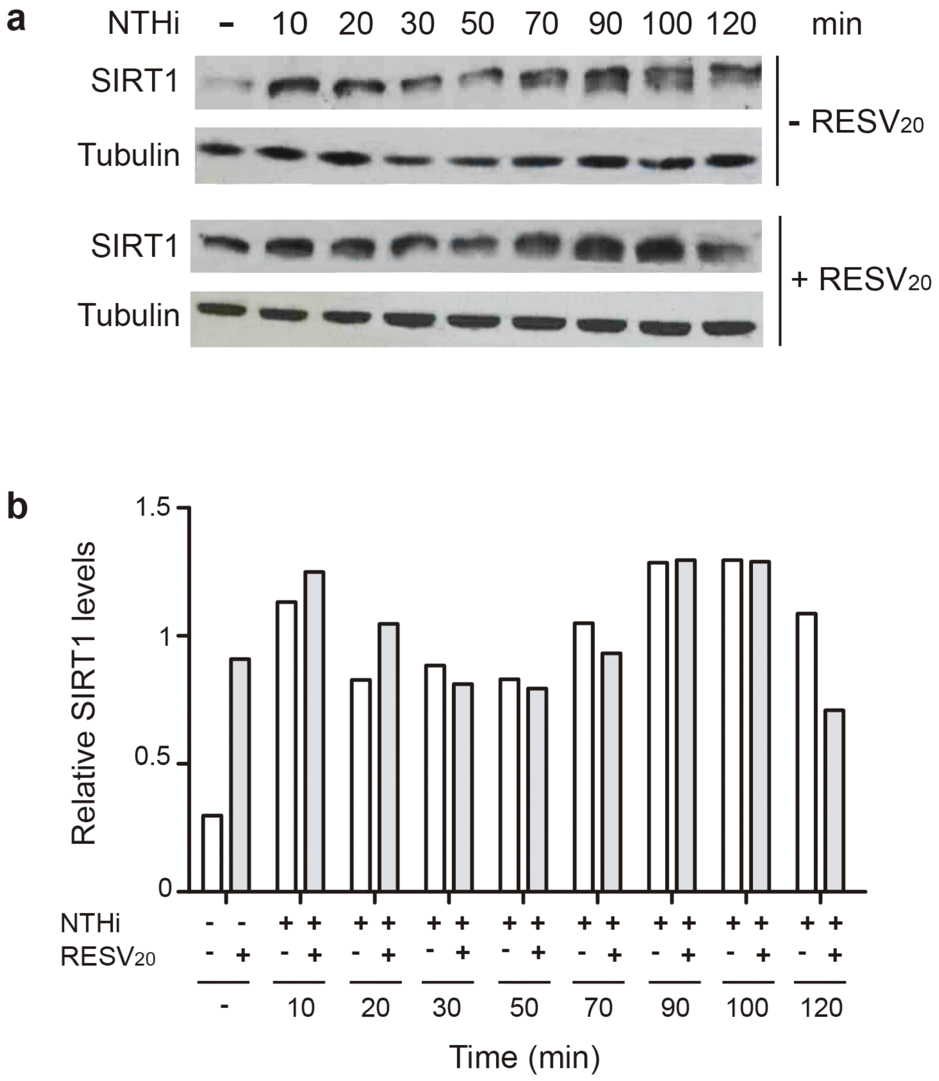

Figure S2. NTHi infection and resveratrol treatment increase SIRT1 protein levels on A549 cells. A549 cells were treated with resveratrol 20 μM for 4 h, and then infected with NTHi375 for 10, 20, 30, 50, 70, 90, 100 or 120 min. **(a)** SIRT1 protein was detected in host cell lysates by western blot analysis with rabbit anti-SIRT1 and goat anti-rabbit IgG antibodies. Tubulin was monitored as a loading control, by using mouse anti-tubulin and goat anti-mouse IgG antibodies. Representative immunoblots are shown. Bands corresponding to SIRT1 (bands ~120 KDa) or tubulin (~55 KDa) were cropped by using Adobe Illustrator CS5. Full-length blots are presented in “Expanded data for Figure S1” (see below). **(b)** Relative level of protein (mean intensity of SIRT1/mean intensity of tubulin) obtained after

densitometry analysis of western blot protein bands at each indicated time point. Please note that samples derive from the same experiment and blots were processed in parallel.

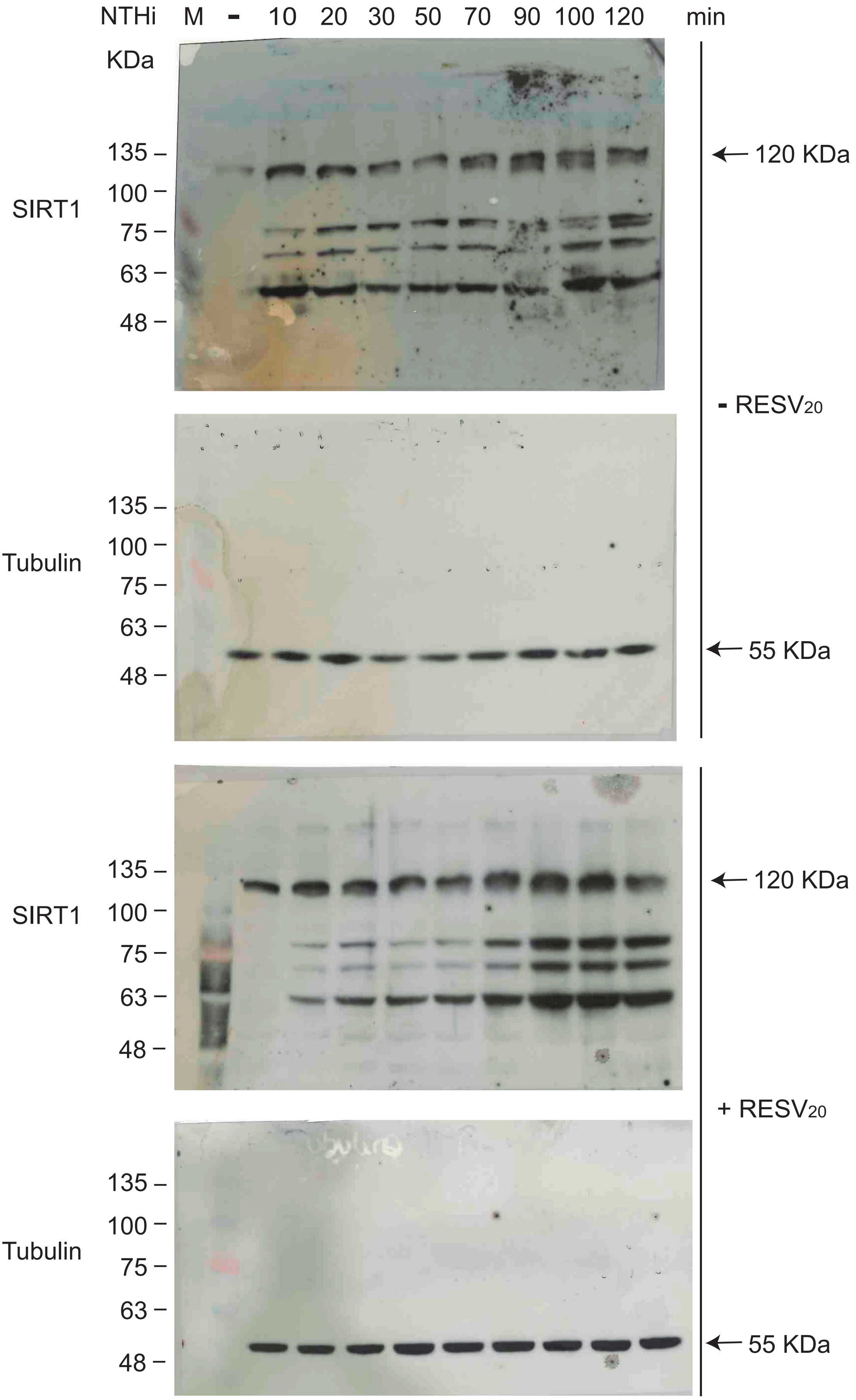
NTHi M - 10 20 30 50 70 90 100 120 min
KDa
SIRT1
135
100
75
63
48
120 KDa
Tubulin
55 KDa
- RESV20
SIRT1
120 KDa
Tubulin
55 KDa
+ RESV20

Expanded data for Figure S2. Full length blots. Protein extracts loaded in the four blots contain samples generated in parallel in the same experiment (both infection time course and non-infected controls). Similarly, samples were processed in parallel, in terms of protein extract preparation, electrophoretic separation and blotting. Each blot was scanned by using a MP C3503 scanner (Ricoh), with a 300 dpi resolution, in color. Scanned images were processed with Adobe Photoshop CS5. Brightness and contrast controlling tools were used across each entire blot to match the color tone of the four images. Bands corresponding to SIRT1 (bands ~120 KDa) or tubulin (~55 KDa) were cropped to improve clarity and conciseness of the presentation in Fig. S1, and combined into one figure with four separate panels (Fig. S1a) by using Adobe Illustrator CS5. Please note that complete experiments were performed at least three times, and blots corresponding to a representative one are shown here. The anti-SIRT1 antibody used in these assays was a rabbit polyclonal antibody raised against amino acids 448-747 of SIRT1 of human origin (sc-15404, Santa Cruz Biotechnology). The anti-tubulin antibody (T5168, Sigma-Aldrich) used in these assays was a mouse monoclonal antibody derived from the B-5-1-2 hybridome; it recognizes an epitope located in the C-terminal end of the α-tubulin isoform.

Table S1. Primers used in this study.

Primer name	Sequence (5´-3´)	Organism	Source
IL8-F-1241	AGAGACAGCAGAGCACAC	Human	[1]
IL8-R-1242	AGTTCTTTAGCACTCCTTGG	Human	[1]
hBD2-F3-1619	TGGCTGAATTCTAACCTCTGTAATGA	Human	This study
hBD2-R3-1620	GGACATCAAGCCTTCCACCTTATA	Human	This study
PDE4B-F-1259	GAGACAAAGAGCGGGAGAGG	Human	This study
PDE4B-R-1260	GGTGGTGAGGGACTTTGAGG	Human	This study
GAPDH-F-1237	GAAGGTGAAGGTCGGAGTC	Human	[1]
GAPDH-R-1238	GAAGATGGTGATGGGATTTC	Human	[1]
KC-F-1404	GACAGACTGCTCTGATGGCA	Mouse	This study
KC-R-1405	TGCACTTCTTTTCGCACAAC	Mouse	This study
mTNFα-F-1592	AGGCACTCCCCCAAAAGATG	Mouse	This study
mTNFα-R-1593	GCTCCTCCACTTGGTGGTTT	Mouse	This study
mGAPDH-F-1430	CCCACTAACATCAAATGGGG	Mouse	[2]
mGAPDH-R-1431	CCTTCCACAATGCCAAAGTT	Mouse	[2]

References

1 Euba, B. *et al*. Genome expression profiling-based identification and administration efficacy of host-directed antimicrobial drugs against respiratory infection by nontypeable *Haemophilus influenzae*. *Antimicrob Ag Chemother* **59**, 7581-7592, doi:10.1128/AAC.01278-15 (2015).

2 Regueiro, V. *et al*. *Klebsiella pneumoniae* subverts the activation of inflammatory responses in a NOD1-dependent manner. *Cell Microbiol* **13**, 135-153, doi:10.1111/j.1462-5822.2010.01526.x (2011).
